# Gas Phase Reactivity of Isomeric Hydroxylated Polychlorinated Biphenyls

**DOI:** 10.1021/jasms.4c00035

**Published:** 2024-04-19

**Authors:** Emma H. Palm, Josefin Engelhardt, Sofja Tshepelevitsh, Jana Weiss, Anneli Kruve

**Affiliations:** † Department of Materials and Environmental Chemistry, Stockholm University, Svante Arrhenius väg 16, 114 18 Stockholm, Sweden; ‡ Luxembourg Centre for Systems Biomedicine (LCSB), University of Luxembourg, 6 avenue du Swing, 4367 Belvaux, Luxembourg; § Department of Environmental Science, Stockholm University, Svante Arrhenius väg 8, 114 18 Stockholm, Sweden; ∥ Institute of Chemistry, University of Tartu, Ravila 14a, 50411, Tartu, Estonia

## Abstract

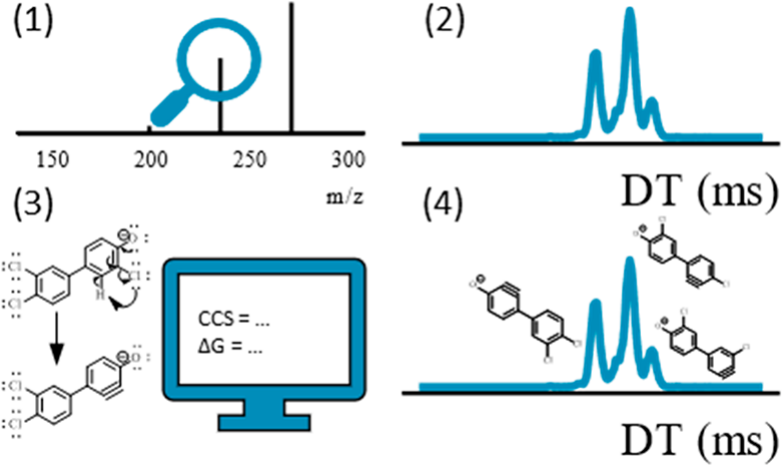

Identification of stereo- and positional isomers detected with high-resolution mass spectrometry (HRMS) is often challenging due to near-identical fragmentation spectra (MS^2^), similar retention times, and collision cross-section values (CCS). Here we address this challenge on the example of hydroxylated polychlorinated biphenyls (OH-PCBs) with the aim to (1) distinguish between isomers of OH-PCBs using two-dimensional ion mobility spectrometry (2D-IMS) and (2) investigate the structure of the fragments of OH-PCBs and their fragmentation mechanisms by ion mobility spectrometry coupled to high-resolution mass spectrometry (IMS-HRMS). The MS^2^ spectra as well as CCS values of the deprotonated molecule and fragment ions were measured for 18 OH-PCBs using flow injections coupled to a cyclic IMS-HRMS. The MS^2^ spectra as well as the CCS values of the parent and fragment ions were similar between parent compound isomers; however, ion mobility separation of the fragment ions is hinting at the formation of isomeric fragments. Different parent compound isomers also yielded different numbers of isomeric fragment mobilogram peaks giving new insights into the fragmentation of these compounds and indicating new possibilities for identification. For spectral interpretation, Gibbs free energies and CCS values for the fragment ions of 4′-OH-CB35, 4′-OH-CB79, 2-OH-CB77 and 4-OH-CB107 were calculated and enabled assignment of structures to the isomeric mobilogram peaks of [M-H-HCl]^−^ fragments. Finally, further fragmentation of the isomeric fragments revealed different fragmentation pathways depending on the isomeric fragment ions.

## Introduction

High-resolution mass spectrometry (HRMS) is widely used for structural identification of chemicals and chemical complexes for novel entities.^1,2^ The identification begins with the high resolution mass spectrum (MS) where the exact mass and isotope pattern are used to obtain the molecular formula of the compound; however, one molecular formula may correspond to thousands of isomeric chemical structures.^3,4^ Thus, the key information used for identification is obtained from the fragmentation spectrum (MS^2^) where the fragment masses and the neutral losses indicate the presense of specific substructures and functional groups.^3^ Based on the MS^2^ spectrum the compounds can be tentatively identified using spectral databases, computational approaches, or manual interpretation. For conclusive identification, the identity of the compound needs to be confirmed with reference standards.^4,5^

Positional isomers and stereoisomers often have very similar MS^2^ spectra, making unequivocal identification of these compounds a challenging task. For instance, Zhang et al.^6^ observed that the fragmentation spectra of positional isomers of epoxides produced from *cis*- and *trans*-hexenol show high overlap. Kasperkowiak et al.^7^ observed a high overlap of the fragmentation spectra of positional isomers of bisphenol F diglycidyl ethers, and Casas-Ferreira et al.^8^ have observed the same for positional isomers of amino acids. This arises as MS^2^ spectra do not reveal the 3D-structural information on such isomeric compounds.

One proposed solution to this problem has been the coupling of ion mobility spectrometry to a high resolution mass spectrometer (IMS-HRMS). IMS enables gas phase separation of ions based on their charge and collision cross section (CCS), which is dependent on the shape and size of the molecule.^9^ IMS-HRMS has been used for identification of isomers of several classes of compounds, such as carbohydrates,^10^ drug metabolites,^11^ and peptides^12^ using the drift time and CCS of the molecular ion. Still, many isomeric parent ions have very similar CCS and remain unresolved in conventional ion mobility separation.^10^ High resolution IMS, accessed via cyclic IMS, has proved useful for such compounds, e.g., PAHs and penta-saccharide isomers.^13,14^

**Figure 1:**
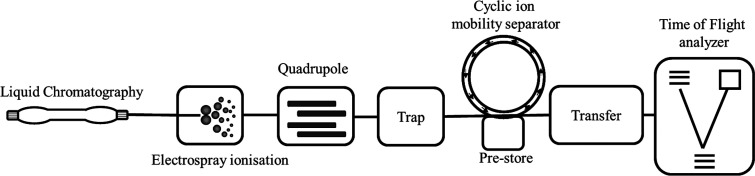
Overview of the cyclic ion mobility mass spectrometer. Fragmentation of the ions may be done in the trap or transfer cells as well as in the prestore and through in-source frgamentation.

In addition, cyclic IMS opens a possibility for gas phase studies of both parent and fragment ions.^15^ This is due to the possibility of separating ions in the IMS and isolating specific peaks of interest based on their drift time in the prestore (Figure 1). From there the ions can be reinjected into the IMS or analyzed with the HRMS. In case of reinjecting the ions into IMS it is possible to further separate the ions by increasing the separation path. Alternatively, it is possible to first activate the isolated parent ions and cause a fragmentation followed by separation of the fragment ions based on ion mobility. Such manipulations may be repeated multiple times, being only limited by the signal intensity that gradually decreases with each separation and fragmentation steps.^15^ Thus, cyclic IMS allows for measuring drift times of parent and fragment ions as well as studying fragmentation pathways.

Recently, Kruve et al.^16^ have shown that the structures of fragment ions formed from isomeric interlocked structures and macrocycles may differ significantly in spite of identical MS spectra, where links, linear, and cyclic fragments could be distinguished using IMS. This allows for following the structural changes occurring during fragmentation rather than comparing the CCS of parent or fragment ions alone. This was further utilized by Lee et al.^17^ who showed that trisaccharides can be distinguished based on the CCS of their fragment ions even if it is impossible to distiguish between the compounds based on the retention times, drift times of the parent ions, and MS^2^ spectra. Small molecules, similarly to macromolecules, undergo fragmentations with structural changes, e.g., ring opening, and different positions of lost functional groups.^18^ Detailed studies of fragmentation pathways of small molecules with IMS-HRMS spectra and drift times for fragment ions are yet to be carried out.

One class of compounds where distinction of isomers is very important is hydroxylated polychlorinated biphenyls (OH-PCBs). These compounds may be formed from polychlorinated biphenyls during metabolism, wastewater treatment, and reactions with oxygen radicals in the atmosphere.^19^ Furthermore, isomeric OH-PCB have different toxic properties.^20^ For instance, OH-PCBs with para-positioned OH-groups and adjacent Cl atoms structurally resemble thyroid hormones and may contribute to thyroid disruption.^19^ Note that we will be referring to the ortho, meta and para positions relative to the carbon atom which binds to the second benzene ring. Recently, we investigated the analytical properties of isomeric OH-PCBs and observed vastly different ionization efficiencies, which makes distinguishing between different isomers important for quantification using electrospray ionization HRMS.^21^

In this study, we use the example of OH-PCBs to investigate how IMS-HRMS can (1) distinguish positional isomers based on their fragment ions and (2) reveal the fragmentation mechanism of positional isomers. To do this the MS^2^ and MS^3^ spectra as well as the drift time of parent and fragment ions were measured for 18 OH-PCBs using cyclic IMS-HRMS. In addition, Gibbs free energies were calculated with density functional theory (DFT) using Gaussian 16 software,^22^ and theoretical CCS values for the potential fragment ions were calculated using IMoS software with the trajectory method^23^ in order to assign tentative fragment ion structures.

## Materials and Methods

### Chemicals

In this study 18 OH-PCB standards were used (Table S1): 4′-OH-CB35, 4′-OH-CB79, 4,4′-diOH-CB80, 4′-OH-CB127, 4,4′-diOH-CB111, 4-OH-CB130, 4-OH-CB193, 4′-OH-CB30, 2-OH-CB77, 4-OH-CB107, 4,4′-diOH-CB83, 4′-OH-CB159, 4-OH-CB172, 4′-OH-CB120, 4,3′-diOH-CB90, 4-OH-CB108, 4,2′-diOH-CB107 and 4,3′-diOH-CB107. All OH-PCB standards were synthesized at Stockholm University, Department of Environmental Chemistry (current Department of Environmental Science), as described by Bergman et al.^24^

For flow injection analysis, the water phase used was prepared from ultra high purity water (Riedel de Haën, HPLC grade, Germany) and ammonium bicarbonate (Sigma-Aldrich, MS grade, Germany). The pH of the buffer was adjusted to 8.0 with ammonia (Merck, 25%, MS grade, Canada). Acetonitrile (Rathburn, S-grade, Scottland) was used as the organic component of the mobile phase.

Theophylline, polyalanine 4–7, sulfaguanidine, sulfadimethoxine, and val-tyr-val all in Major Mix IMS/ToF Calibration Kit (Waters Corporation, U.K.) as well as lauric acid, linolenic acid, cholate (kindly donated by Miklós Mohai from the Stockholm University and Jaanus Liigand from the University of Tartu) were analyzed together with the OH-PCBs for calibration of the cyclic IMS measurements (CCS values vs drift time).

### Instrumental Analysis

All experiments were carried out on an Aquity I Plus UPLC coupled to a Select Series cyclic ion mobility mass spectrometer from Waters Corporation (U.K.) in negative electrospray ionization (ESI) mode. The capillary voltage was 1.8 kV, the source offset was 10 V, and the source and desolvation temperatures were 150 °C and 550 °C, respectively. The cone gas was set to 0 L/hour, and the desolvation gas was set to 800 L/hour. Nebulizer gas was set to 6.0 bar, and the reference capillary was set to 1.5 kV. The instrument was controlled using MassLynx and Quartz (Waters Corporation, U.K.).

For ion mobility separation two pushes per bin with 200 bins in total were used. Racetrack bias was set to 10 V, the repeller was set to 0 V, and the sideways traveling wave (TW) velocity and forward/reverse TW velocity were set to 375 m/s. The TW static height was set to 15 V, the TW start height 15 V, the TW limit height 35 V, and the TW ramping rate was 2.5 V/ms.

All experiments were performed for all 18 OH-PCBs unless otherwise specified. The CCS values were only calculated for the single pass experiments. Furthermore, the number of isomeric fragment peaks may differ for experiments with different numbers of passes since fragments with very similar CCS may appear as a single peak for relatively short path length.

#### Determination of Parent and Fragment Ion CCS

The 18 OH-PCBs were analyzed using flow injections with 20:80 ammonium bicarbonate buffer/acetonitrile mobile phase, flow rate 0.2 mL/min. The monoisotopic molecular ion was isolated in the quadrupole and followed by fragmentation of the OH-PCBs in the trap. Collision voltage ranged between 30 and 40 V depending on the analyte. The cyclic sequence was set to inject for 10.0 ms, separate for 2.0 ms, followed by ejection and acquisition for 26.4 ms. The additional experiments with chromatographic separation are discussed in detail in the Supporting Information (SI).

#### Fragment Ion IMS Separation

To investigate the isomeric fragments, the 18-OH-PCBs were directly infused and fragmented in the ionization source using a cone voltage between 40 and 120 V depending on the OH-PCB which was adjusted to yield the highest signal of the [M-H-HCl]^−^ ion. The fragments were then isolated by the quadrupole with low-mass and high-mass parameters set to 15 and 17 AU, respectively. The fragment ions were then passed 4 cycles around the cyclic ion mobility with separator time from 29.04 to 42.21 ms, depending on the compound.

#### Multidimensional Ion Mobility

To analyze the fragmentation pathways of isomeric fragments, the [M-H-HCl]^−^ fragments were fragmented further using the activation from the prestore to the cyclic ion mobility region. The standards were directly infused to the electrospray source, and the cone voltage was adjusted to yield the highest signal of [M-H-HCl]^−^ and ranged from 40 to 120 V. The *m*/*z* of the fragments were then isolated by the quadrupole with low mass and high mass parameters set to 15 and 17 AU, respectively. The isomeric fragment ions were separated with 4 cycles in the cyclic ion mobility. A single peak of an isomeric fragment ion was ejected to the prestore. The other fragments were ejected, and then stored.

#### Voltage Titration

For two representative compounds, 4′-OH-CB35 and 4-OH-CB107, voltage titration was used to study the voltage required for the formation of the different isomeric [M-H-HCl]^−^ fragments indicating the activation energy required for breaking the respective bonds. These compounds were chosen as they showed more than one [M-H-HCl]^−^ fragment in the previous fragmentation experiments and several possible fragmentation pathways were identified from empirical analysis. The standard solutions of both OH-PCBs were directly infused and the drift times and MS^2^ spectra were recorded at several different trap cell voltages between 6 and 80 V. The fragments were then separated for 4 cycles (27.95 ms) for 4′-OH-CB35 and 6 cycles (57.50 ms) for 4-OH-CB107.

### Data Treatment

Mobilograms and MS^2^ spectra were all obtained using MassLynx version 4.1 (Waters Cooperation, U.K.). The mobilograms were then exported to. csv files for CCS calculations. All computations were performed using R version 4.1.^25^ A Gaussian function was fit to the mobilograms to obtain the drift time (DT) using the nls function with eq 1 and algorithm “port”
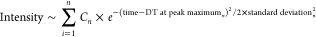
1where *C* is the intensity at the maximum of the mobilogram peak, the time is the measured drift time, and *n* is the number of peaks observed on the mobilogram. As a traveling wave ion mobility instrument the measured DT must be converted to CCS values through a calibration based on known CCS values. The calibration graph was fitted based on 11 compounds with known CCS values (see section Chemicals) from the Unified database developed by McLean research group.^26^ A linear regression (R^2^ = 0.99) was fit between the CCS values and drift time. All CCS values were determined from single pass measurements.

#### Calculations of Ion Properties

The geometry optimization and energy calculations for the potential fragment structures of 4-OH-CB107, 2-OH-CB77, 4′-OH-CB79 and 4′-OH-CB35 were carried out using density functional theory (DFT) M06-2X/6-311+G** method with ultrafine integration grid (implemented in Gaussian 16 software).^22^ Vibrational frequency analysis was carried out to confirm that the results correspond to energy minima. The method was chosen for its ability to calculate conformer energies of anionic species.^27,28^ The optimized geometries were then used to calculate CCS values using the IMoS^23^ trajectory method with induced quadrupole potential enabled and N_2_ gas at 304 K. Partial charges were obtained from natural bond orbital population analysis. Default settings were used for the remaining parameters.

## Results

### MS^2^ Spectra and CCS

The MS^2^ spectra of isomeric OH-PCBs were found to be very similar and the same fragments were observed for all isomeric OH-PCBs, see Figures S2–S8 and Table S1. Most commonly, loss of one or more HCl (all compounds) or loss of CO (14 compounds) were observed. The relative intensities of the formed fragments were also found to be mostly similar. Still, a few fragments showed larger differences. For example, the [M-H-2HCl-CO]^−^ fragment of 4′,4-diOH-CB111 and 4′,2-diOH-CB107 had a relative intensity difference of a factor of 1358, see Figure S6. In addition, in some cases the relative intensity of the fragments of the same *m*/*z* formed from different OH-PCBs was identical, e.g., in case of the [M-H-2HCl-CO]^−^ fragment of 4′,4-diOH-CB111 and 4′,4-diOH-CB83.

Similarly, the CCS values of isomeric OH-PCBs showed only small differences. The minimum difference in measured CCS values was 0.18 Å^2^ (0.1%) and the maximum difference was 2.2 Å^2^ (2.2%), observed for the hexa-chlorinated OH-PCBs and tetra-chlorinated OH-PCBs, respectively. In terms of ion mobility measurements this corresponds to 29 cycles to achieve baseline separation of all hexa-chlorinated OH-PCBs. For the tetra-chlorinated OH-PCBs 6 cycles were required for baseline separation.

**Figure 2:**
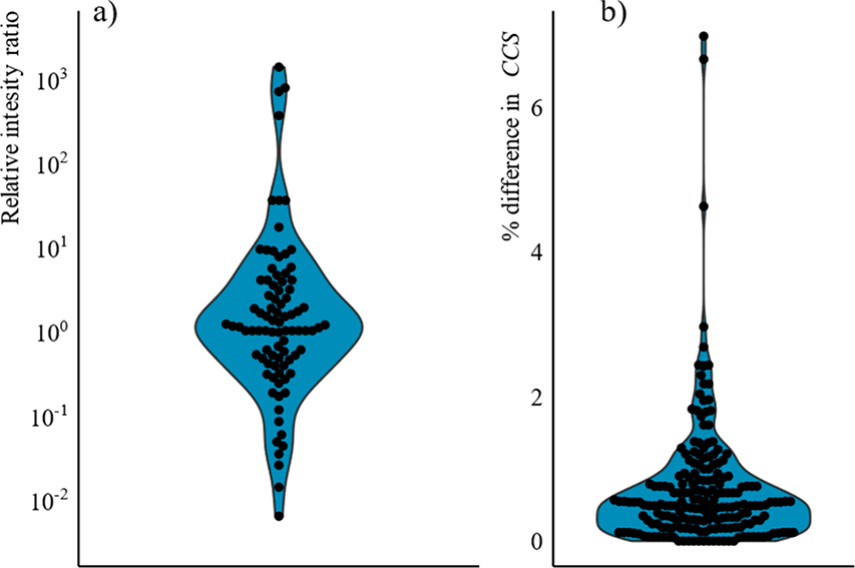
(a) Distribution of differences in relative intensities of fragment ions (calculated as relative intensity of the first fragment ion over relative intensity of second fragment ion). (b) Distribution of the difference in CCS values between isomeric fragments from different parent ions. CCS values were calculated using the single-pass experiments.

In addition, the CCS values of the fragment ions were of interest to study their size and shape. The largest difference in CCS values of these isomeric fragments was 7% observed for the [M-H-2HCl]^−^ ions of 4-OH-CB107 and 4′-OH-CB120; however, most fragments yielded differences of <1% in CCS values, see Figure 2b. Two fragments (the [M-H-2HCl-CO]^−^ fragment of 4,4′-diOH-CB111 and 4,3′-diOH-CB90) had identical CCS values and thus likely identical structures. The CCS values of fragment ions can be found in Tables S2–S8.

**Figure 3:**
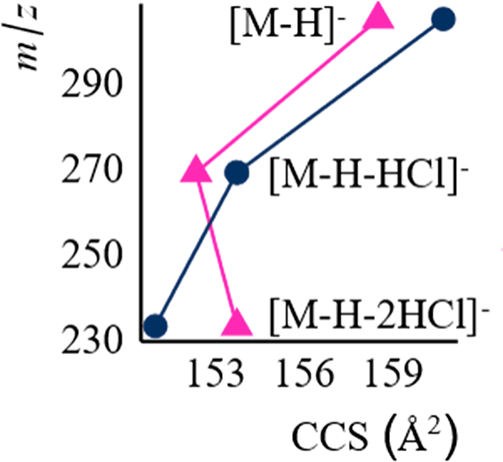
*CCS* values of 4′-OH-CB79 (circles) and 2-OH-CB77 (triangles) as well as their fragments plotted against their *m*/*z*. CCS values were calculated using the single-pass experiments.

Interestingly, in some cases, a parent ion with higher CCS formed a fragment ion with lower CCS than its isomer. One example of this were the tetra-chlorinated OH-PCB isomers. 4′-OH-CB79 has a parent ion with CCS of 160.73 Å^2^ and 2-OH-CB77 of 158.52 Å^2^, while 4′-OH-CB79 yielded a fragment [M-H-2HCl]^−^ of 150.97 Å^2^ and 2-OH-CB77 of 153.73 Å^2^, see Figure 3. Other examples of fragments of lower *m*/*z* having a larger CCS compared to fragments of a higher *m*/*z* include 4,4′-diOH-CB80 and 4′-OH-CB127, see Tables S2 and S8 for their CCS values and Figures S9–S15. This hints that the CCS values of the fragments can indicate significant structural changes occurring during fragmentation. In this case, the increase in CCS most likely originates from ring opening mechanisms as the open structure would be less tight than the ring structure.

### Isomeric Fragment Ions

**Figure 4:**
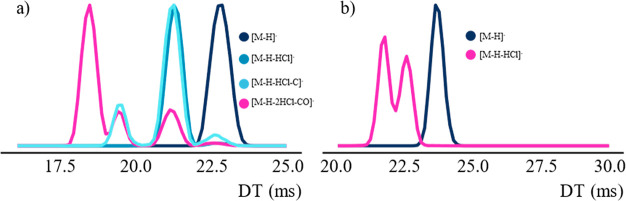
(a) Mobilogram for the [M-H]^−^, [M-H-HCl]^−^, [M-H-HCl-C]^−^, and [M-H-CO-2HCl]^−^ ions of 4,4-diOH-CB80. The intensity has been scaled to the maximum intensity of that fragment for visibility. (b) Mobilogram for the [M-H]^−^ and [M-H-HCl]^−^ ions of 4,3-diOH-CB90. The mobilograms for both compounds were acquired with the method described in the “Determination of parent and fragment ion CCS” section.

After fragmentation many compounds, which were injected separately, yielded multiple peaks on a mobilogram corresponding to the same *m*/*z*. In some cases, these peaks showed a clear overlap with the drift time of a parent ion or heavier fragment ion (Figure 4a) while other fragment peaks were clearly separated from the larger fragments (Figure 4b). Therefore, multiple fragment ion peaks in the mobilogram appeared to have two separate causes. Firstly, peaks overlapping with the peaks of parent ion or larger fragments seemed to result from fragmentation occurring after the ion mobility separation. This could occur due to the acceleration of the ions from the IMS region to the transfer cell. Secondly, multiple fragment ion peaks separated from the peaks of larger fragment ions were hypothesised to indicate isomeric fragment ions formed from the same parent ion.

**Figure 5:**
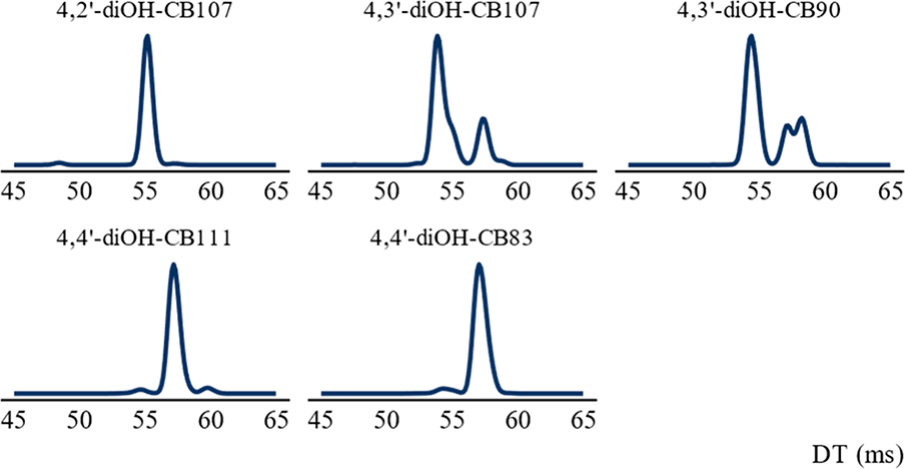
Mobilogram of the dihydroxylated penta-chlorinated OH-PCB [M-H-HCl]^−^ fragments after 4 cycles of ion mobility separation. The *y*-axis shows the relative peak intensity in arbitrary units.

To confirm this hypothesis the compounds were fragmented in-source, followed by filtration of the *m*/*z* of the [M-H-HCl]^−^ fragment in the quadrupole, and ion mobility separation of the formed species. The number of mobilogram peaks for the same *m*/*z* varied from 1 to 4 depending on the OH-PCB. An example can be seen in Figure 5, showing the mobilograms of the [M-H-HCl]^−^ fragments of the dihydroxylated penta-chlorinated OH-PCBs. Some compounds also yielded more mobilogram peaks for the same mass when the number of separation cycles was increased from one to four, indicating a formation of an ensemble of isomeric fragment ions. One example of this was 4′-OH-CB127 which only showed one mobility peak for the [M-H-HCl]^−^ ion after one separation cycle but two peaks after four separation cycles, see Figure S16.

### Fragments Formed from Isomeric [M-H-HCl]^−^ Ions

To further study the fragmentation mechanism of the OH-PCBs, the isomeric [M-H-HCl]^−^ ions were first separated, and one isomer was ejected to the prestore. The ion of interest was then reinjected and simultaneously activated causing fragmentation. Despite low signal intensity and somewhat overlapping [M-H-HCl]^−^ peaks it could be seen that different isomeric [M-H-HCl]^−^ ions formed smaller fragment ions with different *m*/*z* at activation energy 150 V. Here this is illustrated in the form of a fragmentation tree in Figure S7, where the nodes represent the different fragments formed and the edges show which fragments are formed from which precursor through a neutral loss.^29^ An example of this is 4′-OH-CB30 where the [M-H-2HCl]^−^ fragment and [M-H-HCl-CO]^−^ fragment were formed by all three isomeric [M-H-HCl]^−^ ions. Yet, two of the [M-H-HCl]^−^ ions formed [M-H-2HCl-CO]^−^ fragments and [M-H-2HCl-CO-H_2_]^−^ which were not observed for the highest mobility ion. In addition, a fragment ion *m*/*z* of 152.92 Da was observed only for the [M-H-HCl]^−^ ion with second highest mobility. The fragments formed in the further fragmentation of all [M-H-HCl]^−^ ions are show in Table S9.

#### Voltage Titration

**Figure 6:**
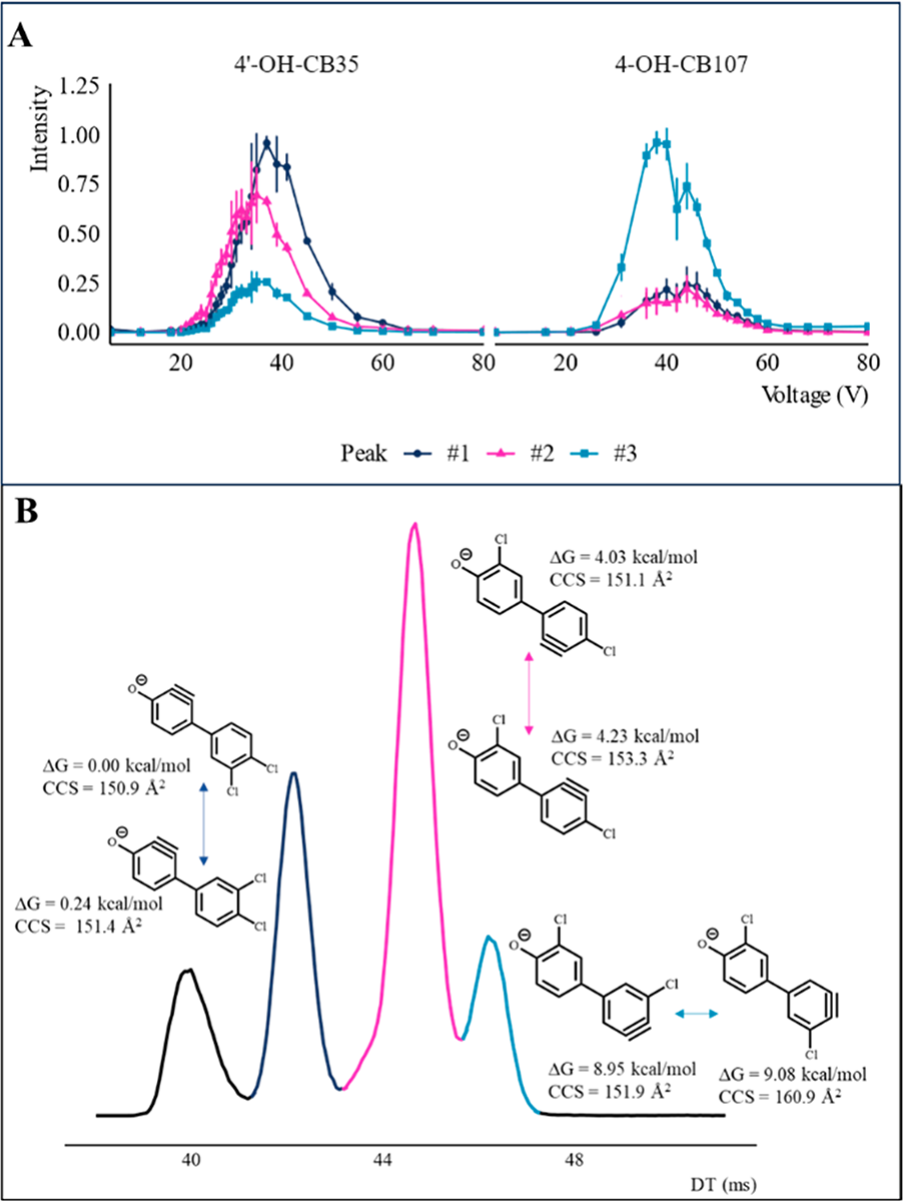
(A) Peak intensity of the different [M-H-HCl]^−^ fragment isomers at all applied trap cell collision voltages for 4′-OH-CB35 and 4-OH-CB107 averaged over three measurements. The error bars indicate the standard deviation. Peak 1 has the shortest drift time and peak 3 has the longest drift time. The normalized intensities at 28 V are approximately 0.18, 0.35, and 0.10 for peaks 1, 2, and 3 respectively. (B) The fragment ion structures assigned to the mobilogram peaks of 4′-OH-CB35 at 28 V based on their calculated CCS as well as obtained peak intensities in the voltage titration. The three fragment peak drift times correspond to four passes around the cyclic IMS. The peak with the shortest drift time corresponds to fragmentation of the parent compound Δ*G* is the calculated free energy relative to the lowest-energy isomer.

4′-OH-CB35 and 4-OH-CB107 were selected for analysis of the stability and determination of CCS of the fragment ions. These were chosen as representatives of the OH-PCBs in this study in terms of the number and position of the chlorine atoms. Also, these OH-PCBs formed multiple isomeric [M-H-HCl]^−^ fragment ions. For these compounds, a voltage titration was used to investigate the activation energy required for the formation of isomeric [M-H-HCl]^−^ fragment ions. For 4′-OH-CB35, the second highest mobility peak (peak 2) starts forming at the lowest voltage (Figure 6A) and remains the dominant peak up to 35 V. The highest mobility isomer (peak 1) starts forming around 18 V and is the highest intensity peak from 35 V onward. The lowest mobility ion (peak 3) had the lowest maximum intensity. For 4-OH-CB107 the lowest mobility peak (peak 3) starts forming first (Figure 6A) and has the highest intensity at all cone voltages. Peaks 1 and 2 both started to form at 26 V and the intensity of peak 1 exceeded the intensity of peak 2.

### Stability and CCS of Fragment Ion Structures

To shed light on the structures of the isomeric fragments the CCS measurements were paired with the DFT calculations. Structures of the parent ions of 4′-OH-CB35 and 4-OH-CB107 were optimized using DFT. The syn and anti conformers of both compounds were found to have similar Gibbs free energy with a difference (Δ*G*) of 0.17 kcal/mol for 4′-OH-CB35 and 0.03 kcal/mol for 4-OH-CB107. In addition, the barrier of interconversion is expected to be low for these compounds. Experimentally, only one mobilogram peak was observed for any of the parent ions of the 18 studied OH-PCBs. These factors combined suggest that syn and anti conformers are easily interconverted.

The calculations were also carried out for the first HCl loss of 4′-OH-CB35, 4′-OH-CB79, 2-OH-CB77, and 4-OH-CB107. The most stable fragment structures for the first HCl loss of 4′-OH-CB35 were the cis/trans isomers formed from the loss of the *meta*-chlorine on the phenolic ring, see Table S10. Similarly, for 4-OH-CB107 the most stable fragments are the ones formed from the loss of either the *meta*-chlorine on the phenolic- or nonphenolic ring. Overall, fragments with a more planar structure had lower Δ*G*, potentially due to an increased charge delocalization. The calculated *CCS* values for 4′-OH-CB35 ranged from 150.9 Å^2^ to 160.9 Å^2^ with the greatest difference being between the trans isomer of the ion resulting from loss of the *meta*-chlorine on the phenolic ring and the cis isomer for the loss of the *para*-chlorine on the nonphenolic ring. For 4-OH-CB107 the calculated CCS values of the [M-H-HCl]^−^ isomers ranged from 157.7 Å^2^ to 160.7 Å^2^. For 2-OH-CB77 the most stable structure for the [M-H-HCl]^−^ fragment was found to contain a 5-membered ring formed with the oxygen and the *ortho-*carbon on the opposite benzene ring, with Δ*G* values over an order of magnitude lower than the other possible fragment structures. The most stable [M-H-HCl]^−^ fragment ion for 4′-OH-CB79 results from the loss of the *meta*-chlorine on the phenolic ring, followed by the loss of the chlorine in the meta position of the nonphenolic ring, and the loss of the chlorine in the para position of the nonphenolic ring. The Δ*G* and CCS values for the most stable [M-H-HCl]^−^ fragments of 4′-OH-CB35, 4′-OH-CB79, 2-OH-CB77 and 4-OH-CB107 can be found in Table S10. For 4′-OH-CB35, all possible HCl losses were investigated. Higher energy fragments, as well as ring opening fragmentations can be seen in Table S11.

## Discussion

The aims of this study were to investigate if cyclic IMS-HRMS could be used to distinguish positional OH-PCB isomers and study their gas phase reactivity. Overall, we found that the approach is promising thanks to the possibility to separate the fragments with multiple IMS cycles. In addition, we were able to find possible mechanisms for the loss of HCl. Below we discuss how the fragment ions can contribute to the identification of isomeric OH-PCBs as well as how the multidimensional IMS-HRMS experiments in combination with DFT calculations can aid the determination of the most likely fragment structures and fragmentation pathways.

### Differences in CCS and Uncertainty

Due to the similarity of the CCS values of the OH-PCB isomers it was impossible to separate them using a single IMS pass. Nevertheless, thanks to the ability to do multiple separation cycles, even the parent ions with as low CCS difference as 0.18 Å^2^ (0.1%) could be (partially) separated after 29 cycles. This demonstrates the benefits of high-resolution ion mobility for the separation of positional isomers of small molecules. In addition, the fragment ions were found to have greater CCS differences than the parent ions in 57% of cases, which shows the benefit in including ion mobility of the fragment ions.

In spite of ion mobility separation of OH-PCBs, the differences in CCS values remain too small to distinguish between the isomers unless the analytical standards are available for confirming the identity. This is due to the uncertainty and error propagation of CCS calculations. To obtain the CCS values of the detected ions with cyclic IMS or other traveling wave ion mobility instruments, a calibration is needed. The calibration accuracy is dependent on the accuracy of the CCS values of the compounds used for calibration, often measured previously with a drift tube instrument.^30^ Generally, the accuracy of reference CCS values is around 2%^15^ and our previous experiences on the same instrument have yielded repeatability standard deviation of 0.35% or better.^31^ In combination with other uncertainty sources the total uncertainty often approaches 3%, making unequivocal identification via database matching or computational CCS approaches complicated if the isomeric candidate structures are expected to have CCS difference below this limit. This occurs though the IMS is able to fully or partially separate ions with even smaller difference in CCS values. Thus, the separation of isomeric chemicals and ability to distinguish between these chemicals without access to reference standards need to be considered independently and latter requires accounting for the uncertainty of the calibration. Furthermore, while the experiments discussed in this article were only performed using a cyclic ion mobility instrument, it could be challenging to distinguish these isomeric compounds and the isomeric fragment ions with ion mobility instruments that have separators with shorter flight paths due to the small difference in CCS values.

### Fragmentation Mechanisms and Isomeric Fragments

To narrow down the possible fragmentation mechanisms, geometries of all possible [M-H-HCl]^−^ fragment ions of 4′-OH-CB35 were optimized with DFT without considering ring-opening, along with a few structures formed by ring opening. Fragments formed from losses of neighboring hydrogen and chlorine atoms had by an order of magnitude lower Gibbs free energies (Δ*G*) than fragment structures formed from loss of non-neighboring chlorine and hydrogen atoms (see Table S11); therefore, the former is considered more likely. Additionally, considering the possibility of ring opening further complicates the analysis of the structure of the fragment ions. For one of the calculated structures formed from a ring-opening the Δ*G* was found to be almost equal to the most stable of the “two-ring structures”, see Table S12. However, this structure was deemed unlikely based on the CCS measurements of the formed fragments as ring opening would result in an increased CCS value relative to the parent ions (Table S10). An example of this was seen for 2-OH-CB77 where the [M-H-2HCl]^−^ fragment has a larger CCS value than the [M-H-HCl]^−^ fragment (Figure 3). Thus, the ring opening mechanism was not further explored for the [M-H-HCl]^−^ ions. Still, the suggested pathway does not fully explain the number of observed peaks for several of the 18 investigated OH-PCBs. For instance, only one mobilogram peak was observed for the [M-H-HCl]^−^ fragment of 4′-OH-CB79, but theoretically 3 different fragmentation sites could be possible based on the Δ*G* values. Based on the calculated CCS there is a possibility of overlapping peaks for the loss of the *meta* and *para*-chlorines on the nonphenolic ring. Still, to fully determine the fragmentation mechanism for this compound, further exploration is needed.

Unambiguous assignment of the number of isomeric fragment ions formed from the same parent ion was complicated as the number of ion mobility peaks varied with the applied cone voltage and number of separation cycles. It is expected that if an ensemble of isomeric ions are formed, more cycles lead to higher resolution and therefore better separation of the ions. However, different fragment ions are likely to require different activation energies, as seen from the voltage titration. This leads to a possibility that the number of isomeric fragments observed is affected by imperfect ion mobility separation or insufficient fragmentation voltage.

### Identification of Potential [M-H-HCl]^−^ Fragment Structures

The [M-H-HCl]^−^ fragment ions were formed for all 18 studied OH-PCBs and the same loss has previously been reported by Li et al.^32^ The calculated CCS of the hypothetical fragment structures, as well as the results of the voltage titration, allow tentative assignment of the structures to the [M-H-HCl]^−^ fragments of 4′-OH-CB35, 4′-OH-CB79, 2-OH-CB77, and 4-OH-CB107. For this purpose, we assume that the fragment structures can rotate between *syn/anti* forms.

In the case of 4′-OH-CB35 the fragment structure with the lowest calculated CCS corresponded to the loss of the *meta*-chlorine on the phenolic ring and was assigned to the peak with the lowest drift time, see Figure 6B. The fragment ion formed by loss of the *meta*-chlorine on the nonphenolic ring was assigned to the second highest mobility peak and the fragment ion formed via cleavage of the *para*-chlorine on the nonphenolic ring was assigned to the lowest mobility peak (Figure 6B). In terms of the voltage titration for the same compound, it was found that the first peak to form was that of the second highest mobility isomer indicating that it likely has the lowest activation energy.

The assignment of the fragment structures to the peaks of 4-OH-CB107 is more ambiguous. Altogether three isomeric [M-H-HCl]^−^ fragment ions were observed with IMS-HRMS and confirmed with voltage titration. In both experiments peaks 2 and 3 could not be fully separated indicating that their CCS values are very similar. In addition, the calculated CCS of the fragments formed from loss of the meta-chlorine on the phenolic or nonphenolic ring were also almost identical. These fragments also have lower calculated CCS values than those arising from the loss of the para-chlorine on the nonphenolic ring. Thus, even assuming that loss of the meta chlorine accounts for the structures of peaks 2 and 3 it will still leave peak 1 unexplained. Therefore, further investigation of the fragmentation mechanism is still needed.

For 2-OH-CB77 only one mobilogram peak was observed for the [M-H-HCl]^−^ fragment ion. While fragmentation mechanisms in collision-induced dissociation are largely controlled by kinetic rather than thermodynamic factors, the calculated energies of the possible fragments suggest that the most probable structure belongs to the fragment forming a 5-membered ring including the oxygen atom (see Table S10), as the energies of the other fragment ions are over 1 order of magnitude higher. Li et al.^32^ have also reported similar fragment structures for less chlorinated OH-PCBs in electron ionization. Similarly, 4′-OH-CB79 also had only one mobilogram peak. However, in this case the differences in Δ*G* between the fragments in the DFT calculation was much smaller than for 2-OH-CB77 making the assignment less certain. Still, the lowest Δ*G* was calculated for the loss of the *meta-*chlorine on the phenolic ring as assuming the thermodynamic stability and the activation energy correlate (which is not certain) this would be the most promising fragment structure.

It is also important to note that the difference between the predicted CCS values of the fragment ions are fairly small and in several cases below the average error of the CCS values predicted by IMoS trajectory model. Thus, contributing to the uncertainty of the structure assignment based on the calculated CCS values. This further highlights the need for more accurate CCS models in order to increase the ability to differentiate between isomeric fragment ions.

### Fragment Fragmentation

**Figure 7:**
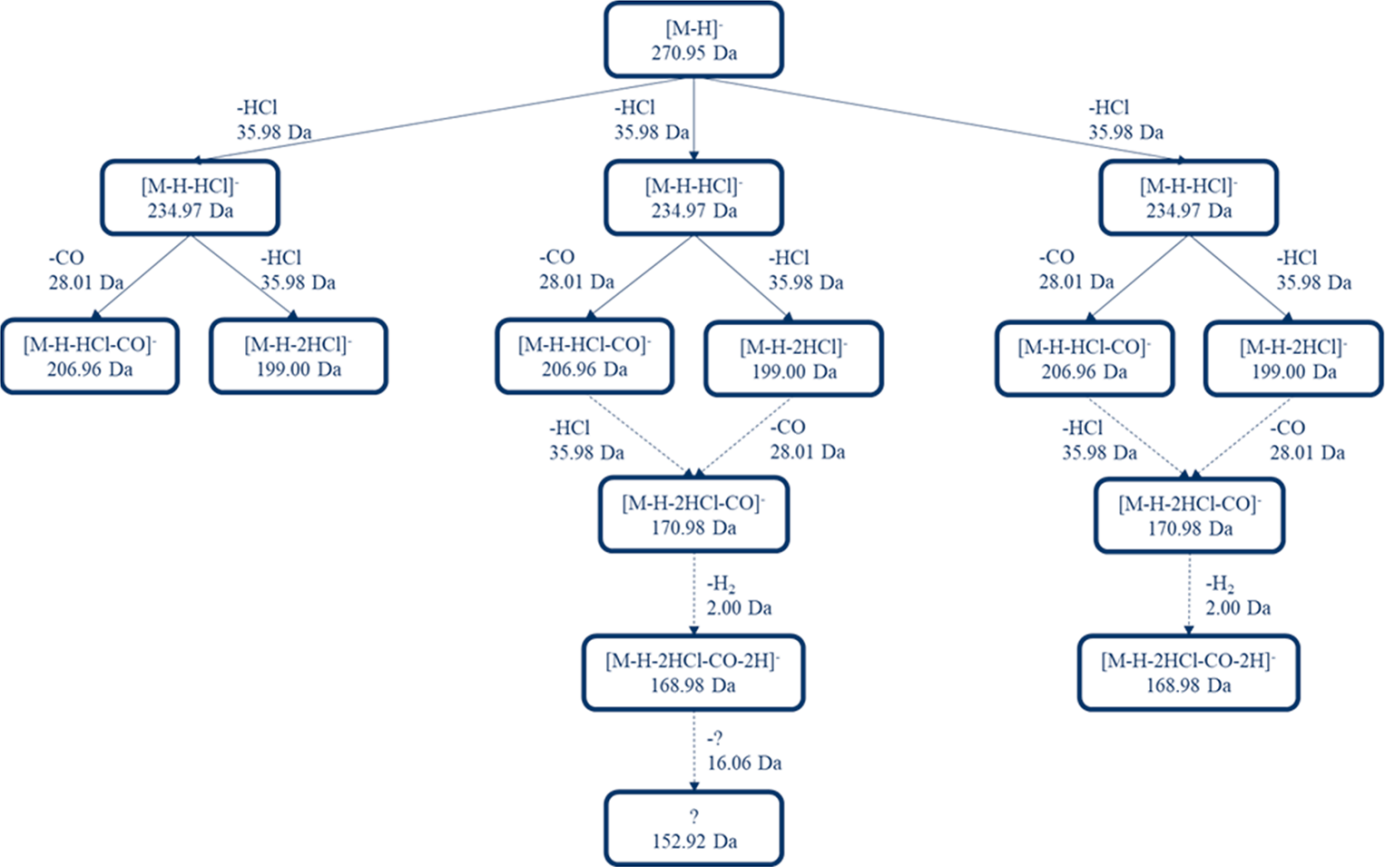
Fragments formed from the isomeric [M-H-HCl]^−^ fragments of 4′-OH-CB30. Dotted lines represent uncertainty in the fragmentation pathway as it is not certain from which of the [M-H-HCL-CO]^−^ or [M-H-2HCl]^−^ the smaller fragments are formed.

To understand the fragmentation mechanism further the [M-H-HCl]^−^ ions were activated in the prestore which led to the second fragmentation event. Interestingly, this resulted in different neutral losses for different isomeric [M-H-HCl]^−^ ions, observed based on the exact mass of the fragments, see Figure 7. The different fragmentation mechanisms of the isomeric [M-H-HCl]^−^ ions are likely to depend on the stability of the first-generation fragment as well as the activation energy used in the fragmentation and the stability of the second-generation fragment.

The presence of different isomeric structures of the same fragment which in turn forms different fragments may also change how fragmentation trees can be used to identify structures and to study fragmentation mechanisms. Currently, fragmentation trees are based on neutral losses, with one molecular formula being present as one node. However, the presence of isomeric fragments which in turn form different smaller fragments suggests that a more accurate representation would be a tree with each node representing a fragment structure which fragments further. This is also used for detailed fragmentation analysis where isomeric fragments have been proposed.^33^

## Conclusions

This study highlights the ability of multipass cIMS experiments for studying the gas phase reactions of isomeric chemicals on the example of fragmentation of 18 OH-PCBs. Cyclic IMS-HRMS shows great promise for the separation and identification of OH-PCBs with the possibility to separate isomers with less than 0.2% difference in CCS. In addition, including ion mobility measurements of the fragment ions proved to be highly useful for distinguishing between OH-PCB isomers as the fragment ions in some cases had greater differences in CCS than the parent ions. Furthermore, the presence of isomeric fragment ions could be confirmed with the number of isomeric fragments varying between different parent compound isomers. The different isomeric fragment ions were also found to follow different fragmentation pathways. We could make tentative structure assignments for the [M-H-HCl]^−^ fragments based on the computational CCS and Δ*G* values in combination with the voltage titration experiment.

## Supporting Information Available

CCS values and ΔG values for parent and fragment ions (XLSX)
Description of liquid chromatography measurements and figures S1–S16 showing chromatograms; MS2 spectra; CCS values of fragment ions and mobilograms for parent and fragment ions of selected compounds (PDF)
Calculated geometries from Gaussian used in CCS predictions (ZIP)
