# Supplementary material for: Gas Phase Reactivity of Isomeric Hydroxylated Polychlorinated Biphenyls
Source: J Am Soc Mass Spectrom. 2024 Apr 19;35(5):1021–9. doi: 10.1021/jasms.4c00035 (PMC11066962; doi:10.1021/jasms.4c00035)
Supplement: Supplementary file 2 — js4c00035_si_002.pdf [file js4c00035_si_002.pdf]

Supporting Information
Gas Phase Reactivity of Isomeric Hydroxylated Polychlorinated Biphenyls

Emma H. Palm[1,2]*, Josefin Engelhardt[3], Sofja Tshepelevitsh[4], Jana Weiss[3], Anneli Kruve[1,3]*

[1] Department of Materials and Environmental Chemistry, Stockholm University, Svante Arrhenius väg 16, 114 18 Stockholm, Sweden
[2] Luxembourg Centre for Systems Biomedicine (LCSB), University of Luxembourg, 6 avenue du Swing, 4367 Belvaux, Luxembourg
[3] Department of Environmental Science, Stockholm University, Svante Arrhenius väg 8, 114 18 Stockholm, Sweden

[4] Institute of Chemistry, University of Tartu, Ravila 14a, 50411 Tartu , Estonia

*emma.palm@uni.lu, anneli.kruve@su.se

Liquid chromatography measurements

For LC analysis a mixed-mode column (Kinetex 2.6 µm PS C18 100 Å, 150 · 3 mm, Phenomenex) with a mixed mode guard column (UPLC PS-C18, 3.0 mm) were used. The water phase used was a pH 8.0 ammonium bicarbonate buffer and the organic phase was acetonitrile. The gradient used was 5% organic modifier which was increased to 90% over 13.5 min where it was kept for 5 min. The organic phase percentage was then lowered back to 5% over 0.1 min. The column was equilibrated 5 min between injections. The flow rate was 0.350 mL/min and the column oven temperature was 30 ˚C. The autosampler temperature was set to 8 ˚C.

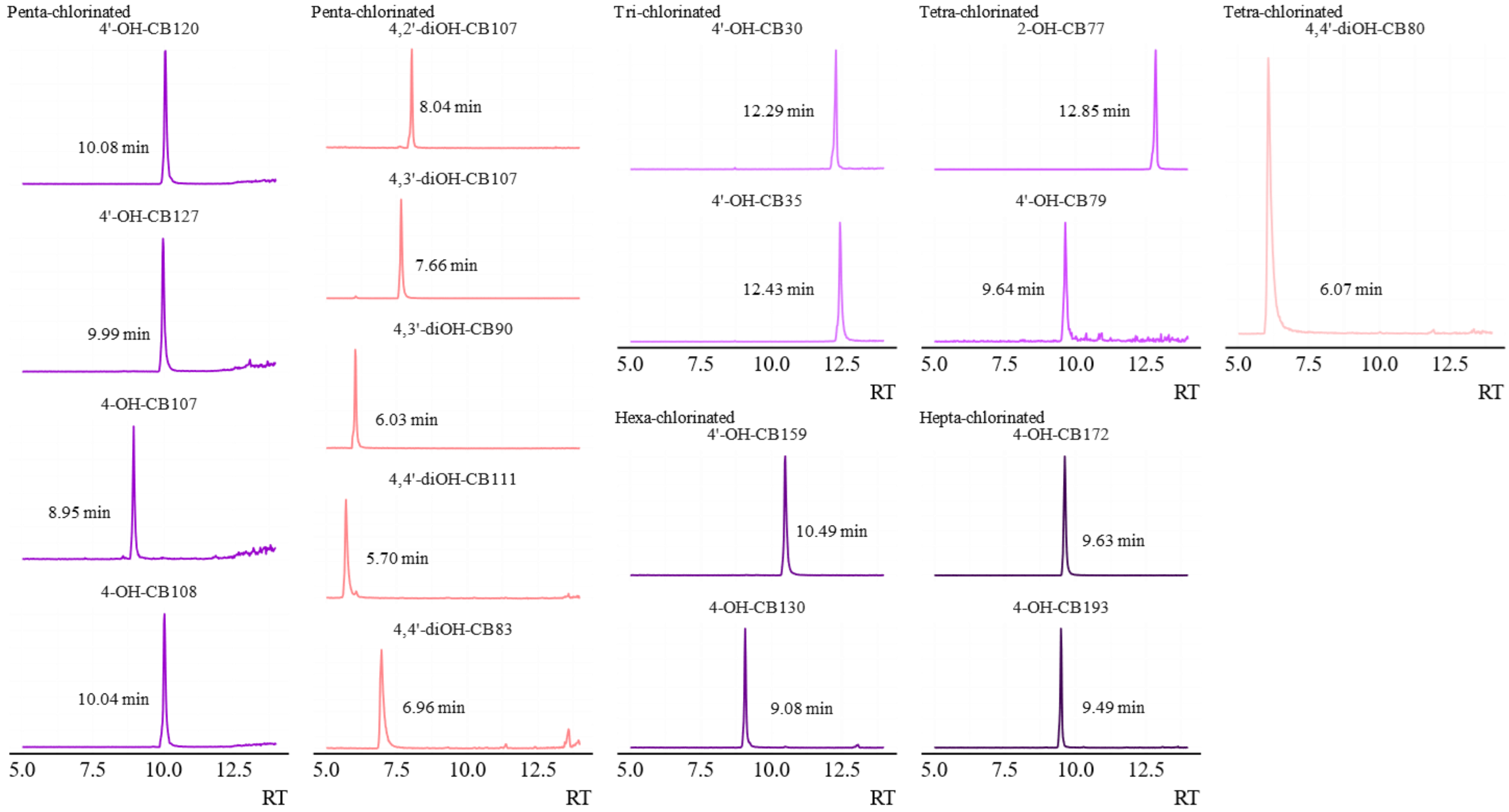

Figure S1 Chromatograms for all OH-PCBs, each group represents a set of isomers. Purple chromatograms belong to mono-hydroxylated compounds and pink chromatograms belong to di-hydroxylated compounds. Darker colours indicate higher degrees of chlorination.

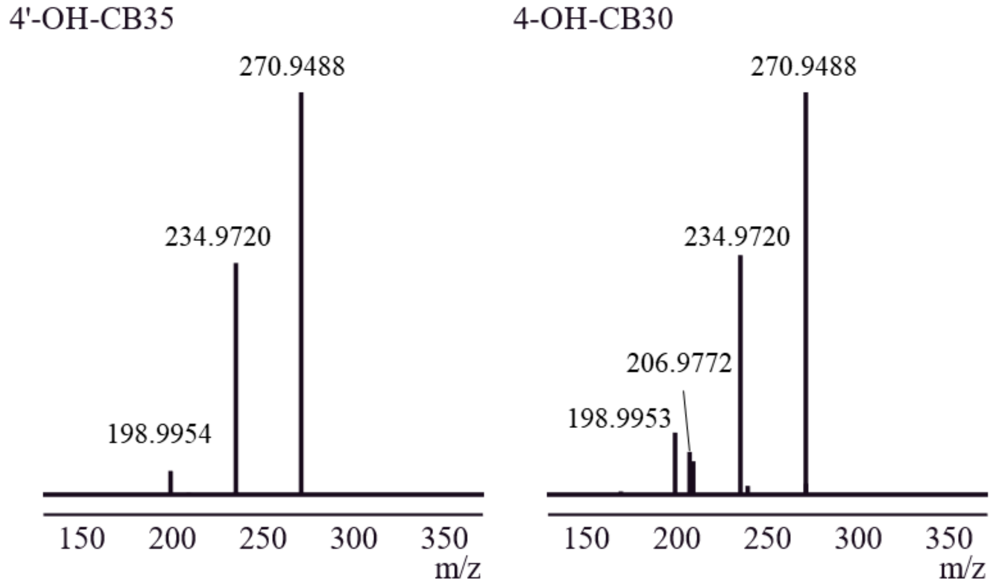

Figure S2 MS^2 spectra of mono-hydroxylated tri-chlorinated OH-PCBs

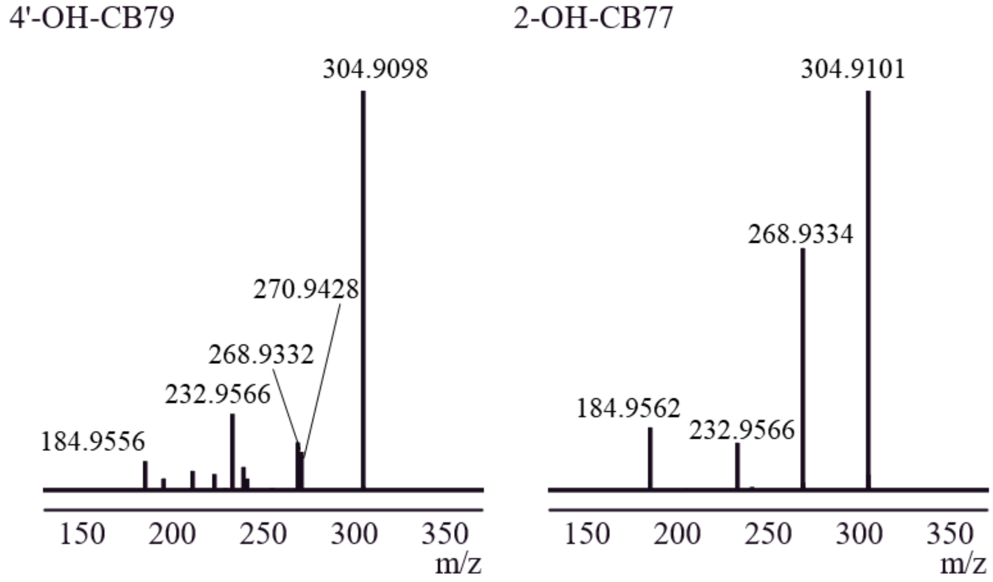

Figure S3 MS^2 spectra of mono-hydroxylated tetra -chlorinated OH-PCBs

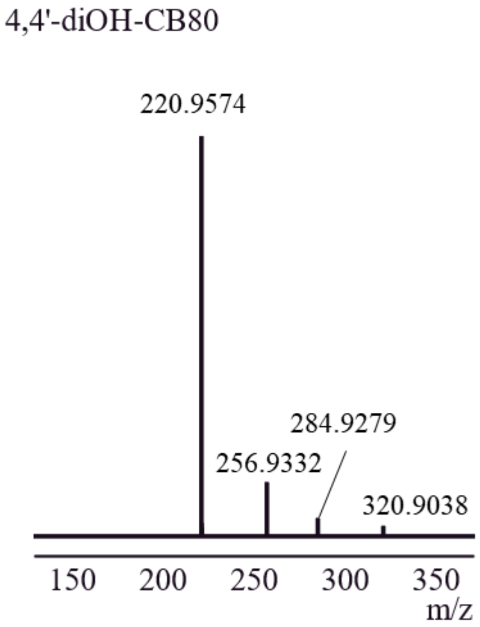

Figure S4 MS[2] spectra of di-hydroxylated tetra -chlorinated OH-PCBs

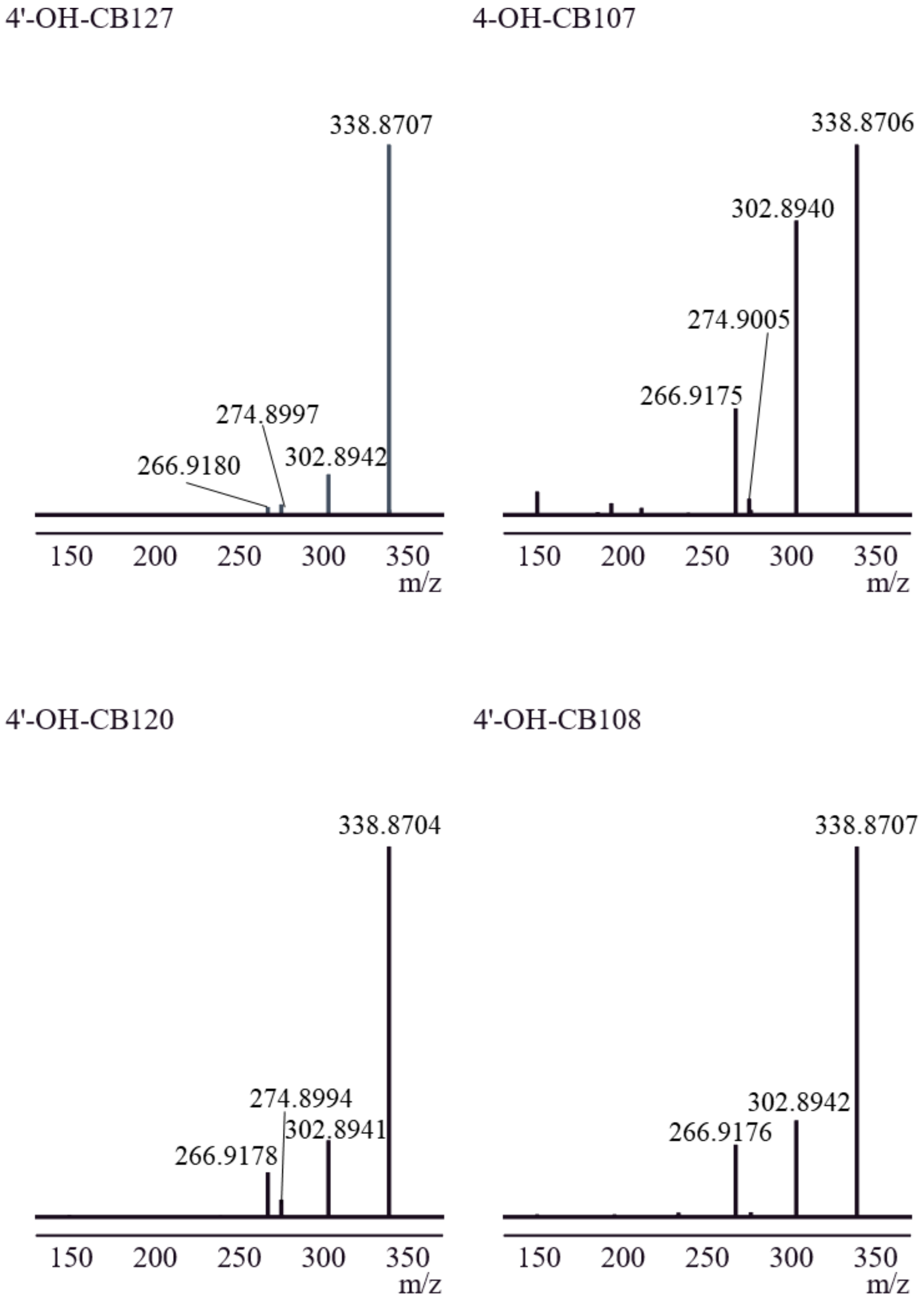

Figure S5 MS^2 spectra of mono-hydroxylated penta-chlorinated OH-PCBs

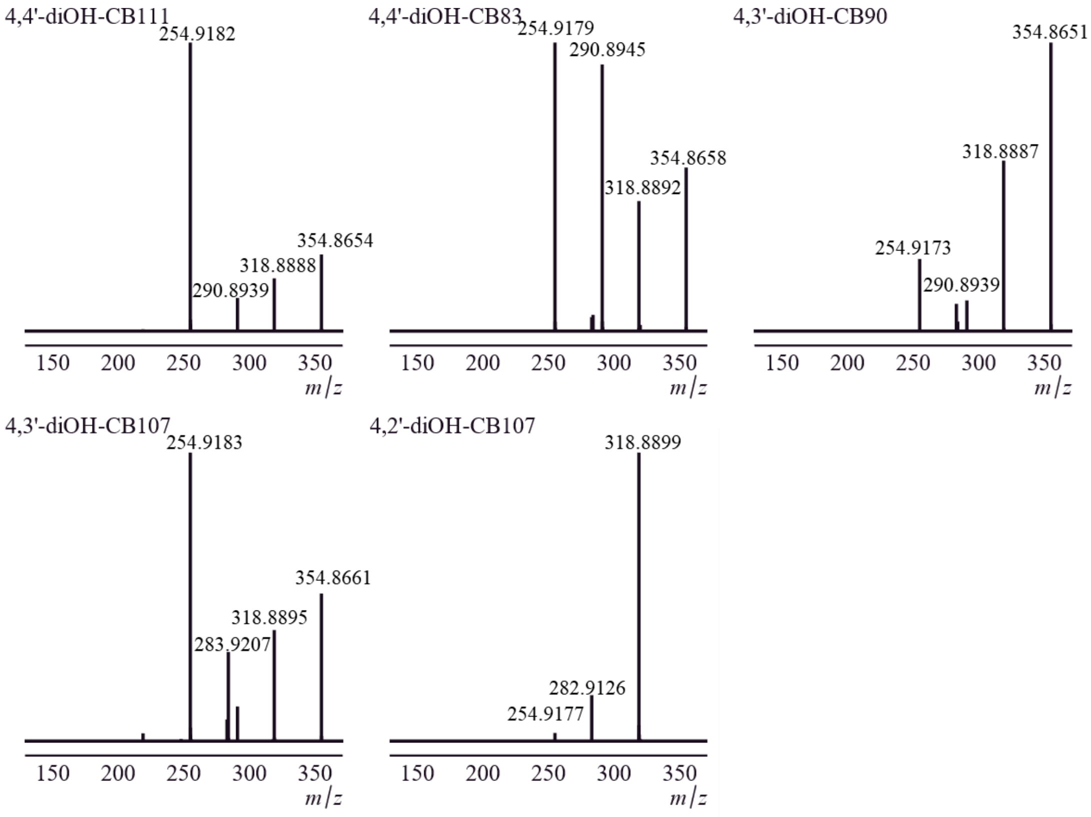

Figure S6 MS[2] spectra of di-hydroxylated penta-chlorinated OH-PCBs

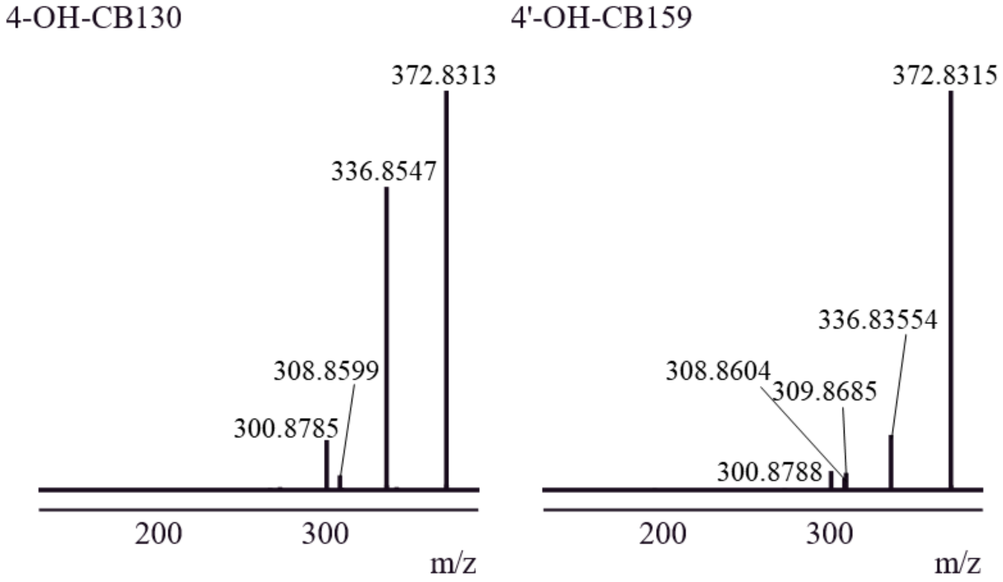

Figure S7 MS[2] spectra of mono-hydroxylated hexa-chlorinated OH-PCBs

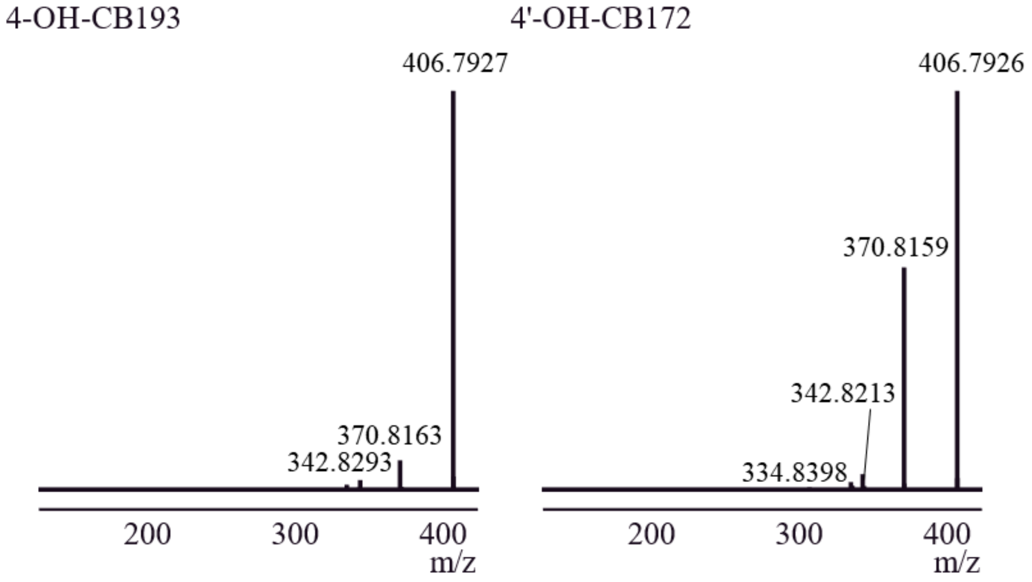

Figure S8 MS^2 spectra of mono-hydroxylated hepta-chlorinated OH-PCBs

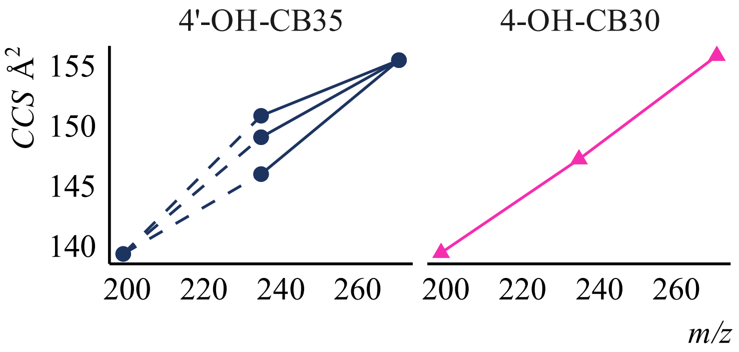

Figure S9 *CCS* values of the tri-chlorinated OH-PCBs and their fragments as a function of their *m/z.* CCS values were calculated using the single-pass experiments.

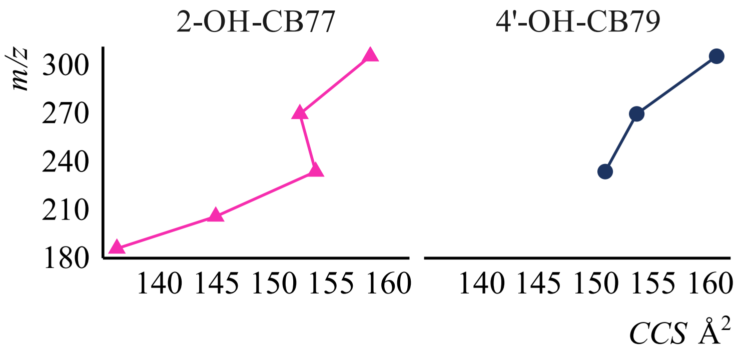

Figure S10 *CCS* values of the mono-hydroxylated tetra-chlorinated OH-PCBs and their fragments as a function of their *m/z.* CCS values were calculated using the single-pass experiments.

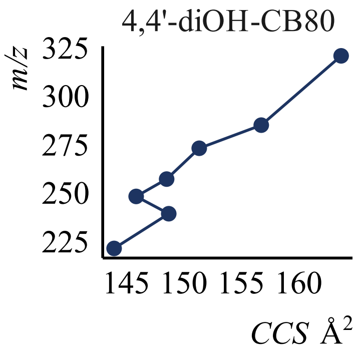

Figure S11 *CCS* values of the di-hydroxylated tetra-chlorinated OH-PCBs and their fragments as a function of their *m/z*. CCS values were calculated using the single-pass experiments.

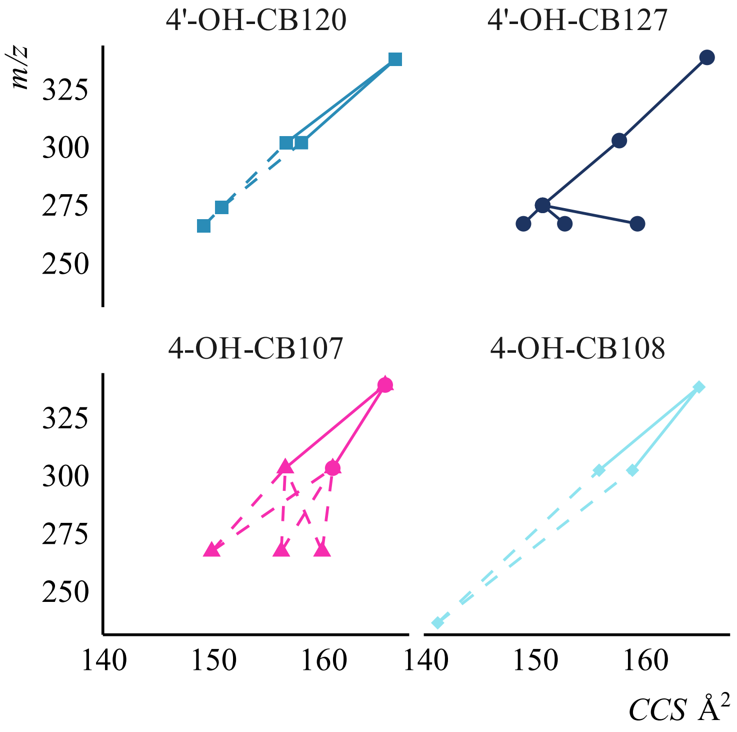

Figure S12 *CCS* values of the mono-hydroxylated penta-chlorinated OH-PCBs and their fragments as a function of their *m/z*. CCS values were calculated using the single-pass experiments.

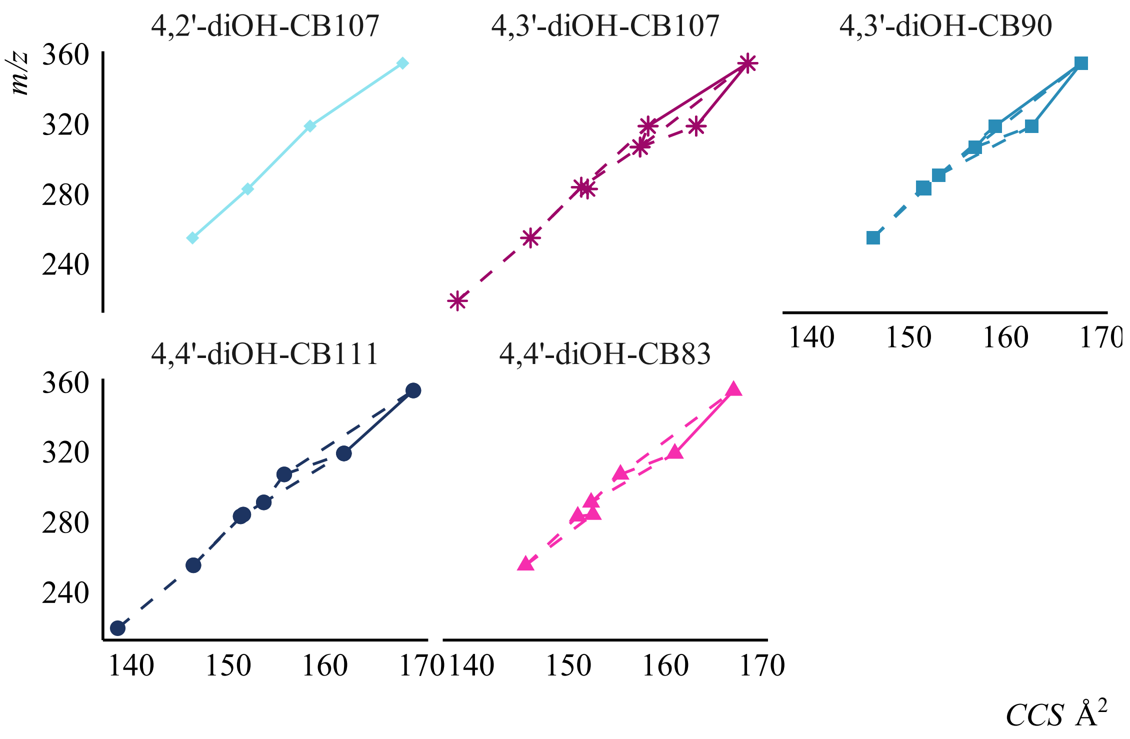

Figure S13 *CCS* values of the di-hydroxylated penta-chlorinated OH-PCBs and their fragments as a function of their *m/z*. CCS values were calculated using the single-pass experiments.

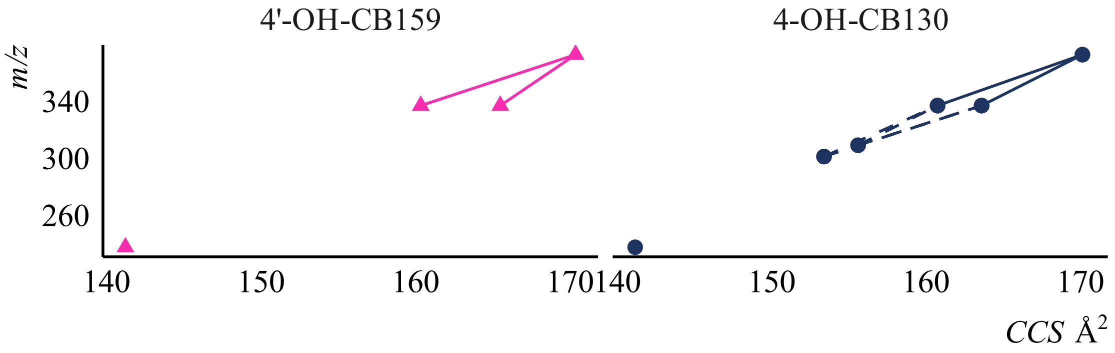

Figure S14 *CCS* values of the hexa-chlorinated OH-PCBs and their fragments as a function of their *m/z*. CCS values were calculated using the single-pass experiments.

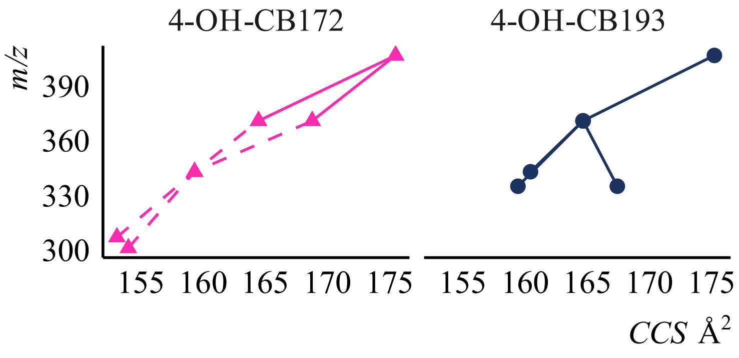

Figure S15 *CCS* values of the hepta-chlorinated OH-PCBs and their fragments as a function of their *m/z*. CCS values were calculated using the single-pass experiments.

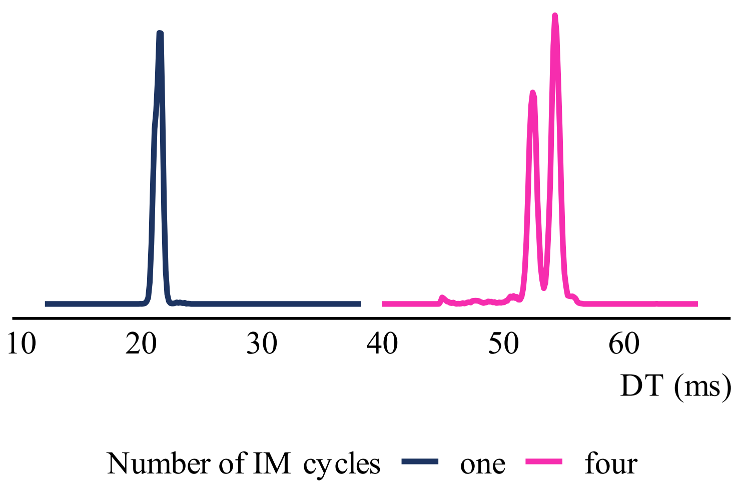

Figure S16 Mobilogram of 4'-OH-CB127 with one and four ion mobility separation cycles of the $[M-H-HCl]^-$ fragment.
